# PReS-FINAL-2173: Protein kinase C delta deficiency is a new cause of monogenic SLE

**DOI:** 10.1186/1546-0096-11-S2-O8

**Published:** 2013-12-05

**Authors:** A Belot, PK Kasher, EW Trotter, AP Foray, AL Debaud, E Meffre, J Brognard, N Bonnefoy, Y Crow

**Affiliations:** 1 Faculty of Human and Medical Sciences, Genetic Medicine, University of Manchester, Manchester, UK; 2 U1111, INSERM, France; 3 Pediatric Nephrology, Rheumatology & Dermatology Department, Hopital Femme Mere Enfant, Hospices Civils de Lyon, Lyon, France; 4 Paterson Institute for Cancer Research, Cancer Research UK, University of Manchester, Manchester, UK; 5 Department of Immunobiology, Yale University, New Haven, USA; 6 IRCM, Institut de Recherche en Cancérologie de Montpellier, U896, INSERM, Montpellier, France

## Introduction

Systemic lupus erythematosus (SLE) is a prototype autoimmune disease. Infectious triggers, genetic background, immunological abnormalities and environmental factors are all supposed to interact in disease development. Rare causes of monogenic SLE have been described, (e.g. complement deficiencies, interferonopathies and FasL deficiency) providing unique insights into fundamental mechanisms of immune tolerance.

## Objectives

Our objective was to identify the cause of an autosomal recessive form of SLE in an inbred family with three affected siblings.

## Methods

We investigated three siblings and used next generation sequencing to identify mutations in the disease-associated gene. We performed extensive biochemical, immunological and functional assays to assess the impact of the identified mutations on B cell biology.

## Results

Genetic mapping and targeted exome sequencing lead to the identification of a homozygous mutation in PRKCD, encoding protein kinase C delta (PKCδ). Mutation of PRKCD resulted in reduced expression and activity of encoded protein PKCδ. In mouse, PKCδ plays a crucial role in the deletion of autoreactive B cells. As for mice deficient in PKCδ, we demonstrated that B cells display a resistance to calcium-dependent apoptosis and a higher proliferation rate associated with an increase of immature B cells in affected patients, and a developmental shift toward an immature phenotype of naïve B cells.

## Conclusion

Our findings indicate that PKCδ is crucial in regulating B cell tolerance and preventing self-reactivity in humans.

## Disclosure of interest

None declared.

